# Differential impact of deep intraparenchymal hyperdensities versus subarachnoid/cortical hyperdensities on futile reperfusion after mechanical thrombectomy: a propensity score-matched study

**DOI:** 10.3389/fneur.2025.1686898

**Published:** 2025-12-17

**Authors:** Meijuan Dong, Yanfeng Wang, Bo Sun, Ruoyu Zhou, Kun An, Mingchao Li

**Affiliations:** 1 Department of Endocrinology, The Affiliated Huaian No.1 People’s Hospital of Nanjing Medical University, Huaian, China; 2 Department of Neurology, The Affiliated Huaian No.1 People’s Hospital of Nanjing Medical University, Huaian, China; 3 Medical College, Shandong University of Traditional Chinese Medicine, Jinan, China

**Keywords:** acute ischemic stroke, mechanical thrombectomy, futile reperfusion, deep intraparenchymal hyperdensities, subarachnoid/cortical hyperdensities

## Abstract

**Background:**

Despite successful mechanical thrombectomy (MT), futile reperfusion (FR) remains a major challenge in acute ischemic stroke (AIS). While post-MT hyperdense areas (HDAs) on non-contrast computed tomography (CT) are associated with reperfusion injury, the differential effects of anatomical HDA subtypes—deep intraparenchymal (DIH) versus subarachnoid/cortical (SCH)—on FR risk are unclear.

**Methods:**

We retrospectively analyzed 864 AIS patients undergoing MT (2017–2023). HDAs detected within 0.5 h post-MT were classified as DIH or SCH. Propensity score matching (PSM) balanced baseline confounders (1:1 DIH: SCH). Risk factors for FR were analyzed using a multivariate logistic regression analysis in the PSM cohort.

**Results:**

After PSM, 116 patients in the DIH group were matched with 116 patients in the SCH group. In total, 91 patients (78.5%) in the SCH group and 72 patients (62.1%) in the DIH group suffered FR (*p* = 0.006). A multivariate analysis showed that SCH significantly increased the risk of FR (OR: 3.103, 95%CI: 1.425–6.759, *p* = 0.004), indicating that patients with SCH have a 3.103 times higher risk of FR than patients with DIH.

**Conclusion:**

Anatomical HDA subtypes differentially predict FR risk, with SCH portending a worse prognosis. This subtype classification enables early risk stratification and may guide personalized post-MT management.

## 1 Introduction

Acute ischemic stroke (AIS) has become a devastating disease with high mortality and morbidity (1). Currently, endovascular treatment, particularly mechanical thrombectomy (MT), has emerged as the first-line therapy for patients with large-vessel occlusion (2). Randomized controlled trials have shown that MT dramatically improves functional outcomes (3, 4). Nonetheless, a considerable number of patients (41%–55%) treated with MT fail to achieve a favorable outcome at 3 months, despite successful reperfusion, which is known as futile reperfusion (FR) (5–7).

While successful MT significantly improves outcomes in AIS patients with large-vessel occlusion, reperfusion injury remains a critical determinant of clinical prognosis. Hyperdense areas (HDAs) on non-contrast CT (NCCT) post-thrombectomy have been recognized as potential imaging markers of reperfusion injury (8, 9), but their underlying pathophysiological heterogeneity is poorly characterized. Previous studies have typically analyzed HDAs as a homogeneous entity (10–12), potentially overlooking distinct mechanistic and clinical implications associated with their anatomical distributions—particularly between deep intraparenchymal hyperdensities (DIH) and subarachnoid/cortical hyperdensities (SCH). DIH is hypothesized to arise from microvascular injury (e.g., blood–brain barrier disruption and compromised integrity), while SCH may relate to extensive reperfusion. These mechanistic distinctions, inferred from cerebrovascular pathophysiology, require further direct validation (13, 14). This discrepancy suggests that anatomical subtype classification may provide a more precise imaging biomarker for prognostic assessment.

To investigate the heterogeneous effects of different anatomical subtypes of HDAs on FR risk, this study categorizes HDAs into two subtypes—DIH and SCH—based on anatomical location and compares their FR risk using propensity score matching (PSM) to control for confounding factors. We hope to establish a clinically actionable framework for early risk stratification of HDAs (DIH versus SCH), which may guide personalized perioperative decision-making in neurointerventional practice.

## 2 Materials and methods

### 2.1 Subjects

**Figure 1:**
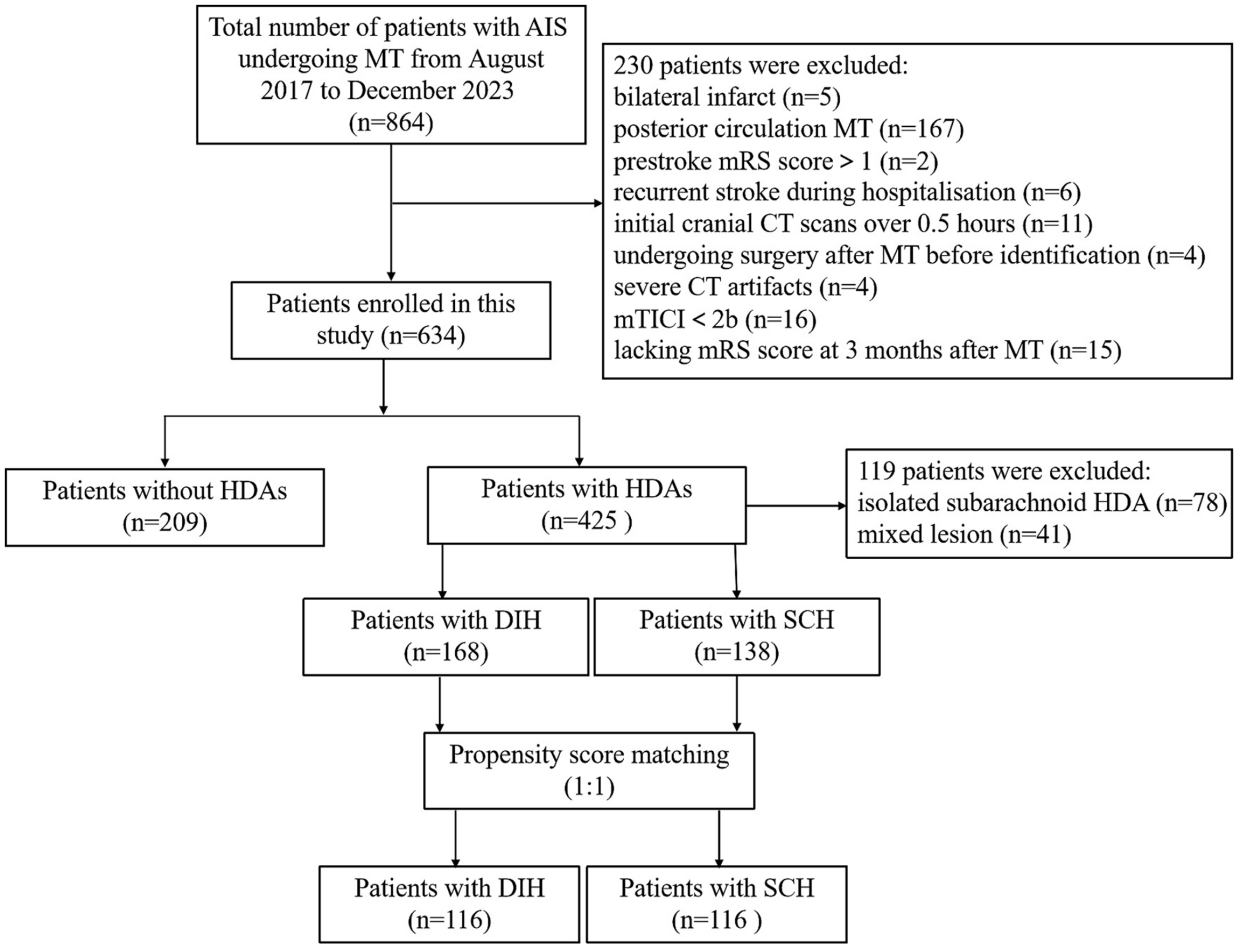
Flowchart of the inclusion and exclusion criteria. AIS, acute ischemic stroke; MT, mechanical thrombectomy; mTICI, modified thrombolysis in cerebral infarction score; mRS, modified Rankin scale; HDAs, hyperdense areas; DIH, deep intraparenchymal hyperdensities; SCH, subarachnoid/cortical hyperdensities.

A total of 864 patients with AIS underwent MT in our hospital from August 2017 to December 2023. The selection of the study population is shown in Figure 1. This retrospective study was approved by the Ethics Committee of Huaian No.1 People’s Hospital, and the requirement for patient informed consent was waived (approval number KY-2023-046-01). The inclusion criteria were as follows: (1) patients aged ≥ 18 years; (2) patients with AIS with unilateral anterior circulation large artery occlusion undergoing MT; (3) patients with successful reperfusion with modified thrombolysis in cerebral infarction (mTICI) score at level 2b-3; (4) patients with a cranial NCCT scan within 0.5 h after MT; and (5) patients with a modified Rankin scale (mRS) score assessed at 3 months after MT. The exclusion criteria were as follows: (1) patients with bilateral infarcts; (2) patients undergoing posterior circulation MT; (3) patients with prestroke mRS score>1; (4) patients with recurrent stroke during hospitalization; (5) patients whose initial cranial CT was performed >0.5 h after MT; (6) patients who had surgery after EVT, prior to identification of HDAs; (7) patients with severe CT artifacts; (8) patients with mTICI<2b; and (10) patients lacking mRS score at 3 months after MT.

### 2.2 Clinical data collection

The following clinical data were collected, including demographics (age and gender), vascular risk factors (smoking, alcohol consumption, atrial fibrillation, diabetes mellitus, hypertension, hyperlipidemia, coronary heart disease, and previous stroke), admission systolic and diastolic blood pressure (SBP and DBP), baseline National Institutes of Health Stroke Scale (NIHSS) score, Alberta Stroke Program Early Computed Tomography (ASPECT) score, etiology of stroke (large artery atherosclerosis, cardioembolism, stroke of other determined etiology, and stroke of undetermined etiology), occlusion site (internal carotid artery and middle cerebral artery), and endovascular therapy information (onset to puncture time, puncture to reperfusion time, onset to reperfusion time, cases of thrombolysis, number of thrombectomy, and postoperative mTICI score).

Clinical data were extracted from the hospital information system by experienced and well-trained clinicians in accordance with standard care protocols, following approval by the Institutional Review Board and in compliance with national data protection regulations. All data were obtained from routine clinical practice, and the data collection process did not alter standard clinical procedures. To ensure data security and patient confidentiality, all information was encoded, de-identified, and securely transmitted. Data quality was further enhanced by identifying and managing invalid formats and extreme outliers, thereby ensuring the completeness and accuracy of the dataset.

### 2.3 Mechanical thrombectomy

All enrolled patients were selected according to stroke guidelines and underwent MT under local anesthesia by two neurointerventionists with 10 years of experience. MT was performed using stent retrievers (Solitaire AB [Covidien/ev3, Irvine, USA] and Solitaire FR [Covidien/ev3, Irvine, USA]) or react suction devices (Covidien/ev3, Irvine, USA). The physician performing the neurointervention regularly reported the number of stent retriever passes. If targeted arterial recanalization failed, rescue therapies such as stent implantation, balloon angioplasty, intracatheter tirofiban administration, or intra-arterial thrombolysis would be used.

### 2.4 Definition and classification of DIH and SCH

HDAs were defined as focal or patchy abnormal imaging regions on non-enhanced head computed tomography (NCCT) images obtained immediately after the procedure (typically within 0.5 h), with a CT value > 50 HU and a density visibly higher than that of the surrounding normal brain parenchyma. All NCCT images were evaluated using standardized window settings: window width (WW): 90 HU and window level (WL): 30–35 HU.

**Figure 2:**
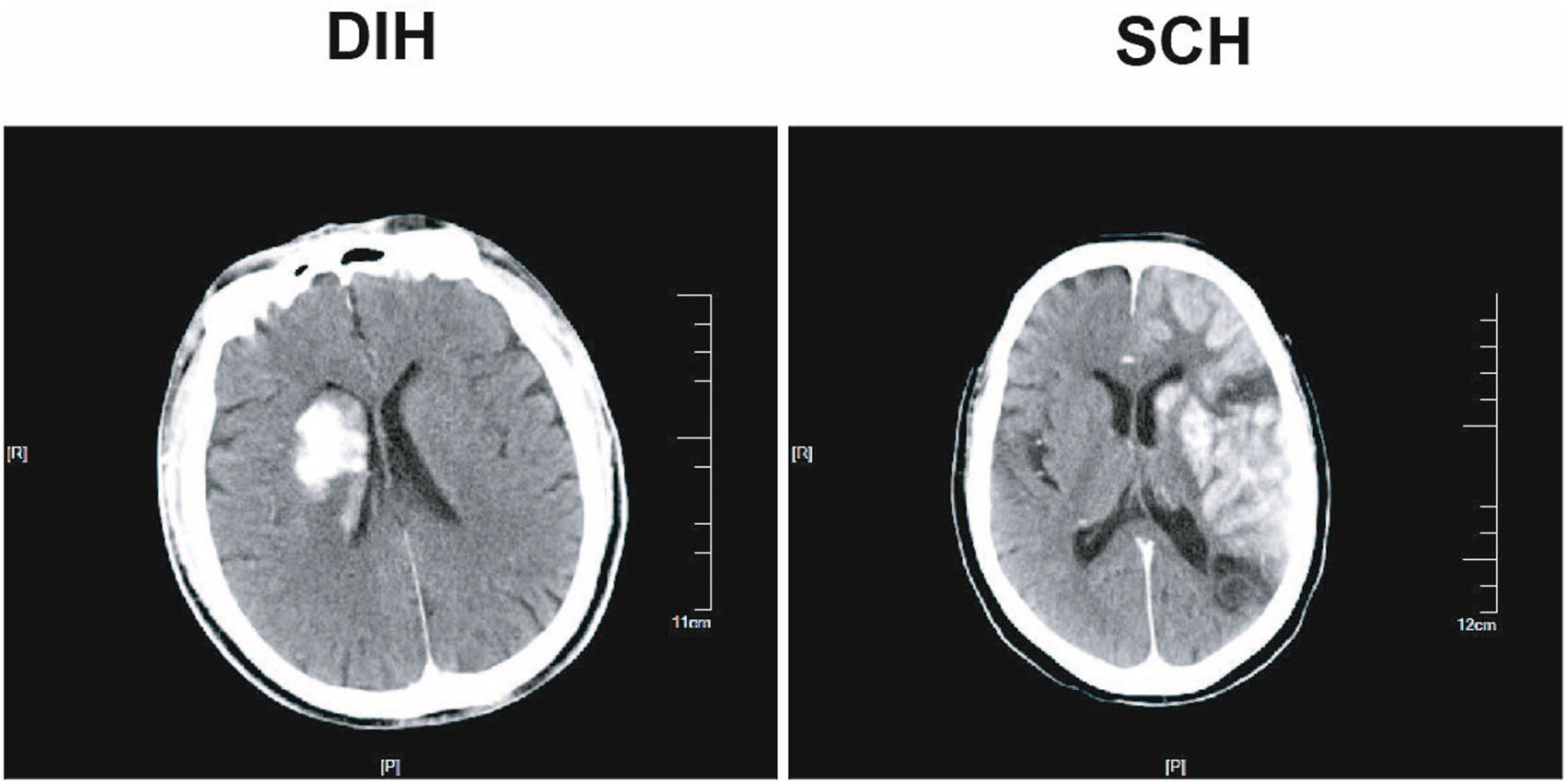
DIC and SCH on cranial NCCT images within 0.5 h after MT. DIH, deep intraparenchymal hyperdensities; SCH, subarachnoid/cortical hyperdensities; NCCT, non-contrast computed tomography; MT, mechanical thrombectomy.

DIH was defined as new HDAs localized to the deep brain parenchyma, without involvement of the subarachnoid space, ventricles, or other non-parenchymal regions, as observed on NCCT images within 0.5 h after MT. SCH was defined as new HDAs involving both the subarachnoid spaces (including the Sylvian fissure, cerebral convexity, interhemispheric fissure, or perimesencephalic cisterns) and cortical structures, as observed on NCCT images within 0.5 h after MT (Figure 2).

If both DIH- and SCH-type HDAs were present together, the lesion was classified as the predominant type (e.g., DIH or SCH) when the volume proportion of that type was ≥ 80%, as estimated using the 3D Slicer software. If the proportion of either type was < 80%, the lesion was classified as a mixed lesion and excluded from the analysis.

The CT images were analyzed by two experienced radiologists who were blinded to the clinical outcomes, patient identities, and group assignments. To assess the reliability of image-based classifications, inter-rater reliability was evaluated using the intraclass correlation coefficient (ICC) based on initial independent assessments. Intra-rater reliability was assessed by having each radiologist re-evaluate a randomly selected subset of cases (*n* = 30) after an interval of approximately 2 weeks, with the goal of evaluating consistency over time. Any discrepancies in classification between raters or across time points were resolved through consensus discussion.

### 2.5 Assessment of reperfusion efficacy

Patients who achieved successful reperfusion after MT (mTICI at level 2b-3) and had a favorable functional outcome at 3 months were classified as having good reperfusion (mRS score ≤ 2). Patients who achieved successful reperfusion after MT but had an unfavorable outcome (mRS score ≥ 3) were classified as having futile reperfusion.

### 2.6 Statistical analysis

A statistical analysis was performed using SPSS 27.0 software and R software (version 4.2.3). The missing data were handled using multiple imputation based on the chained equations algorithm, generating five complete datasets. The imputation model incorporated all study variables and potential covariates influencing the missingness mechanism. To assess the impact of imputation on data reliability, a sensitivity analysis was performed by evaluating the internal consistency of the scale using Cronbach’s alpha coefficient (*α* ≥ 0.7 considered acceptable). The coefficient was calculated separately for each of the five imputed datasets, followed by computation of the mean, standard deviation, and confidence interval. The results demonstrated good internal consistency, with minimal variation in the coefficients across the datasets, indicating that multiple imputation did not significantly compromise data reliability.

PSM analysis was used to balance variables between the groups. The PSM was performed using the nearest neighbor matching method at a 1:1 ratio to match the DIH group and the SCH group with a caliper distance of 0.02. In our study, variables that showed a significant difference between the two groups in the univariate analysis were used to create a propensity score for each subject, including ASPECT score, puncture to reperfusion time (PRT), and onset to reperfusion time (ORT). No significant collinearity was observed among the above variables. In addition, although there were no differences in demographics (age and gender) between the groups, we also matched these two variables. The standardized mean difference (SMD) was used to assess the efficacy of the matching. An SMD of less than 0.1 was considered to be a negligible difference. To assess the robustness of our findings, we conducted sensitivity analyses using inverse probability of treatment weighting (IPTW) to account for potential confounding and treatment assignment biases.

Continuous variables conforming to the normal distribution were expressed as mean ± S.E.M., and the two-tailed *t*-test was used for comparison between different groups. Variables of skewed distribution were described by the median (interquartile range), and the non-parametric Mann–Whitney U-test was used to compare differences. For categorical variables presented as frequency and percentage, the chi-square test or Fisher’s exact test was used to analyze differences between the groups. Multivariate logistic regression analysis estimated the odds ratio (OR) and 95% confidence interval (CI) for each variable (DIH was selected as the reference variable in the final multivariate analysis). It was assumed that no collinearity existed between variables included in the multivariate logistic regression analysis if the tolerance (the tolerance range was 0–1) was greater than 0.1 and the variance inflation factor was less than 10. A *p*-value of <0.05 was considered statistically significant.

## 3 Results

### 3.1 Prematch comparison of the DIH group versus the SCH group

**Figure 3:**
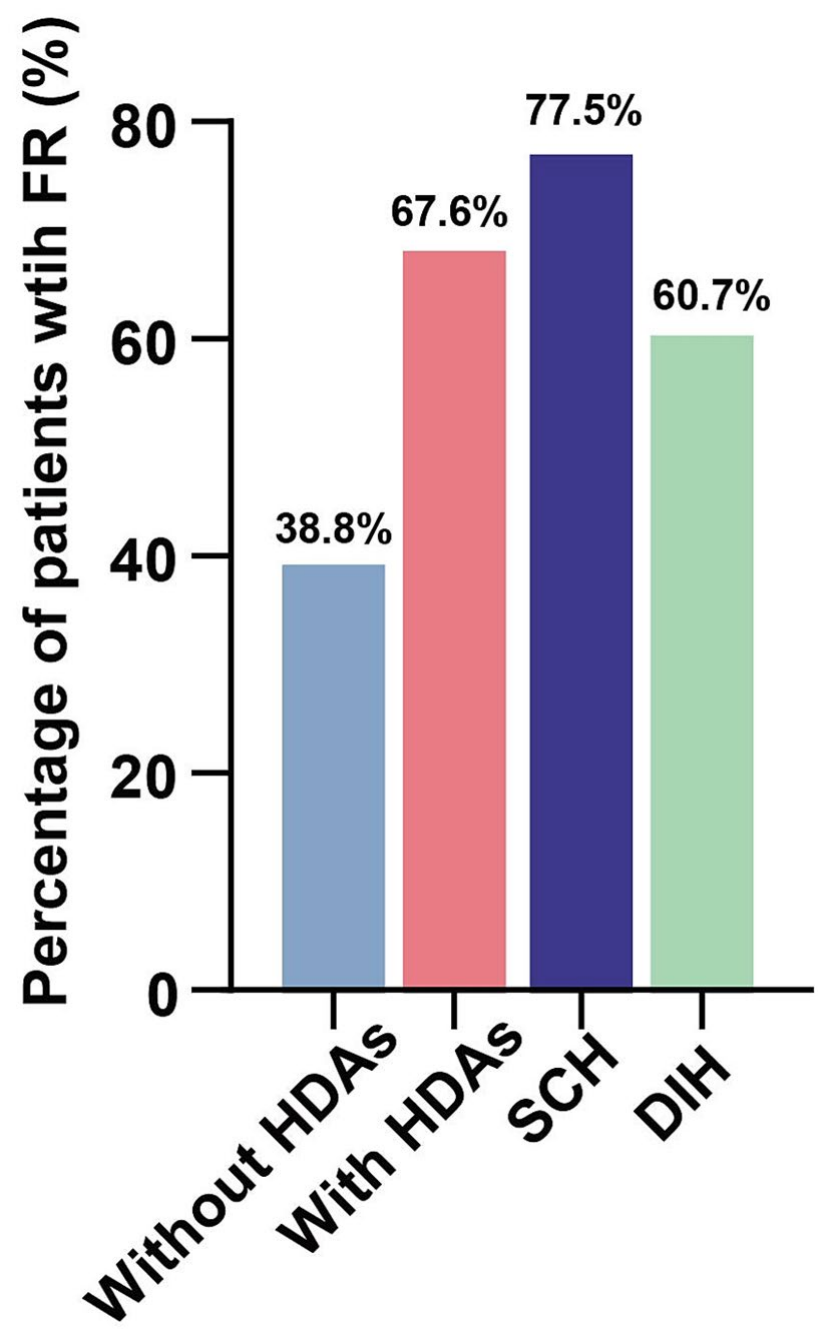
Incidence of futile reperfusion in patients with and without HDAs. FR, futile reperfusion.

A total of 864 patients with AIS underwent MT, and 230 patients were excluded according to the exclusion criteria (Figure 1). We analyzed the incidence of FR in 634 patients, including 425 patients with HDAs and 209 patients without HDAs. The results showed that the incidence of FR was significantly higher in patients with HDAs (67.6%) than in patients without HDAs (38.8%). Among the patients with HDAs (excluding 78 patients with isolated subarachnoid HDAs and 41 patients with mixed lesions), 168 (39.5%) patients had DIH and 138 (32.5%) had SCH. There was a significant difference in the incidence of FR between the two types of HDAs, 60.7% for DIH and 77.5% for SCH, and both were higher than in patients without HDAs (Figure 3).

Subsequently, 306 patients with DIH or SCH were matched between the DIH group and the SCH group. The median age of all patients was 69.5 (63–77) years, and 164 (53.6%) patients were male. Before matching, we compared the clinical characteristics between the two groups (Table 1). The results showed that there was a significant difference between the two groups in the following three variables: ASPECT score, PRT, and ORT.

**Table 1:** Clinical characteristics of patients with HDAs before and after PSM.

Variables	Before PSM			After PSM		
	DIH (*n* = 168)	SCH (*n* = 138)	*P*-value	DIH (*n* = 116)	SCH (*n* = 116)	*P*-value
Demographic data						
Age (years)	68 (63–77)	71.5 (63–77)	0.464^a^	69.68 ± 11.65	69.38 ± 10.89	0.839^c^
Male, *n* (%)	98 (58.3)	66 (47.8)	0.067^b^	57 (49.1)	61 (52.6)	0.599^b^
Vascular risk factors, *n* (%)						
Diabetes mellitus	34 (20.2)	39 (28.3)	0.101^b^	27 (23.3)	32 (27.6)	0.451^b^
Hypertension	111 (66.1)	80 (58)	0.145^b^	77 (66.4)	67 (57.8)	0.176^b^
Hyperlipidemia	18 (10.7)	16 (11.6)	0.807^b^	13 (11.2)	13 (11.2)	1.000^b^
Atrial fibrillation	95 (56.5)	84 (60.9)	0.381^b^	72 (62.1)	69 (59.5)	0.575^b^
Coronary heart disease	16 (9.5)	15 (10.9)	0.698^b^	9 (7.8)	13 (11.2)	0.370^b^
Previous stroke	13 (7.7)	8 (5.8)	0.504^b^	11 (9.5)	8 (6.9)	0.473^b^
Smoking	38 (22.6)	31 (22.5)	0.974^b^	24 (20.7)	28 (24.1)	0.529^b^
Alcohol consumption	39 (23.2)	29 (21)	0.645^b^	29 (25)	26 (22.4)	0.643^b^
Stroke etiology, *n* (%)			0.925^d^			0.614^d^
LAA	67 (39.9)	52 (37.7)		40 (34.5)	45 (38.8)	
CE	97 (57.7)	83 (60.1)		74 (63.8)	69 (59.5)	
Others	4 (2.4)	3 (2.2)		2 (1.8)	2 (1.8)	
Occlusive site, *n* (%)			0.212^b^			0.395^b^
ICA	59 (35.1)	60 (43.5)		48 (41.4)	58 (50.0)	
MCA	96 (57.1)	72 (52.2)		68 (58.6)	58 (50.0)	
Admission baseline data						
SBP (mmHg)	149.72 ± 24.50	149.86 ± 24.78	0.960^c^	148.14 ± 24.53	150.27 ± 24.55	0.509^c^
DBP (mmHg)	84.74 ± 14.91	85.15 ± 14.06	0.807^c^	84.1 ± 15.76	85.17 ± 14.08	0.586^c^
NIHSS score	22 (18–26)	24 (18–27.25)	0.130^a^	22 (18–26)	24 (18–27)	0.279^a^
ASPECT score	8 (7–9)	9 (8–9)	0.022^a^	8 (7–9)	8 (8–9)	0.319^a^
MT treatment procedure						
Thrombolysis, *n* (%)	36 (21.4)	34 (24.6)	0.506^b^	24 (20.7)	27 (23.3)	0.634^b^
Number of thrombectomy passes	2 (1–3)	2 (1–2)	0.910^a^	2 (1–3)	2 (1–3)	0.667^a^
OPT (min)	294.58 ± 115.95	281.68 ± 108.19	0.319^c^	280.03 ± 95.11	294.29 ± 109.45	0.291^c^
PRT(min)	81.5 (59.25–120)	66 (50–113)	0.048^a^	70 (50–100)	75 (50–120)	0.588^a^
ORT (min)	390 (330–442.25)	362 (302.5–430.25)	0.013^a^	384 (330–415.25)	381 (316–437.75)	0.694^a^
mTICI score	5 (4–5)	5 (4–5)	0.706^a^	5 (4–5)	5 (4–5)	0.661^a^

Values are listed as frequency (percentage), mean ± SD, or median (interquartile range). *P*-value of “a” represents the Mann–Whitney U-test, “b” represents the chi-square test, “c” represents Student’s *t*-test, and “d” represents Fisher’s exact test. HDAs, hyperdense areas; DIH, deep intraparenchymal hyperdensities; SCH, subarachnoid/cortical hyperdensities; PSM, propensity score matching; SBP, systolic blood pressure; DBP, diastolic blood pressure; NIHSS, National Institute of Health Stroke Scale; ASPECT, Alberta Stroke Program Early Computed Tomography Score; LAA, large artery atherosclerosis; CE, cardioembolism; ICA, internal carotid artery; MCA, middle cerebral artery; OPT, onset to puncture time; PRT, puncture to reperfusion time; ORT, onset to reperfusion time; mTICI, modified thrombolysis in cerebral infarction. For statistical analysis, the mTICI score was numerically encoded as follows: 0 = 1, 1 = 2, 2a = 3, 2b = 4, and 3 = 5.

### 3.2 Propensity-matched comparison of the DIH group versus the SCH group

**Figure 4:**
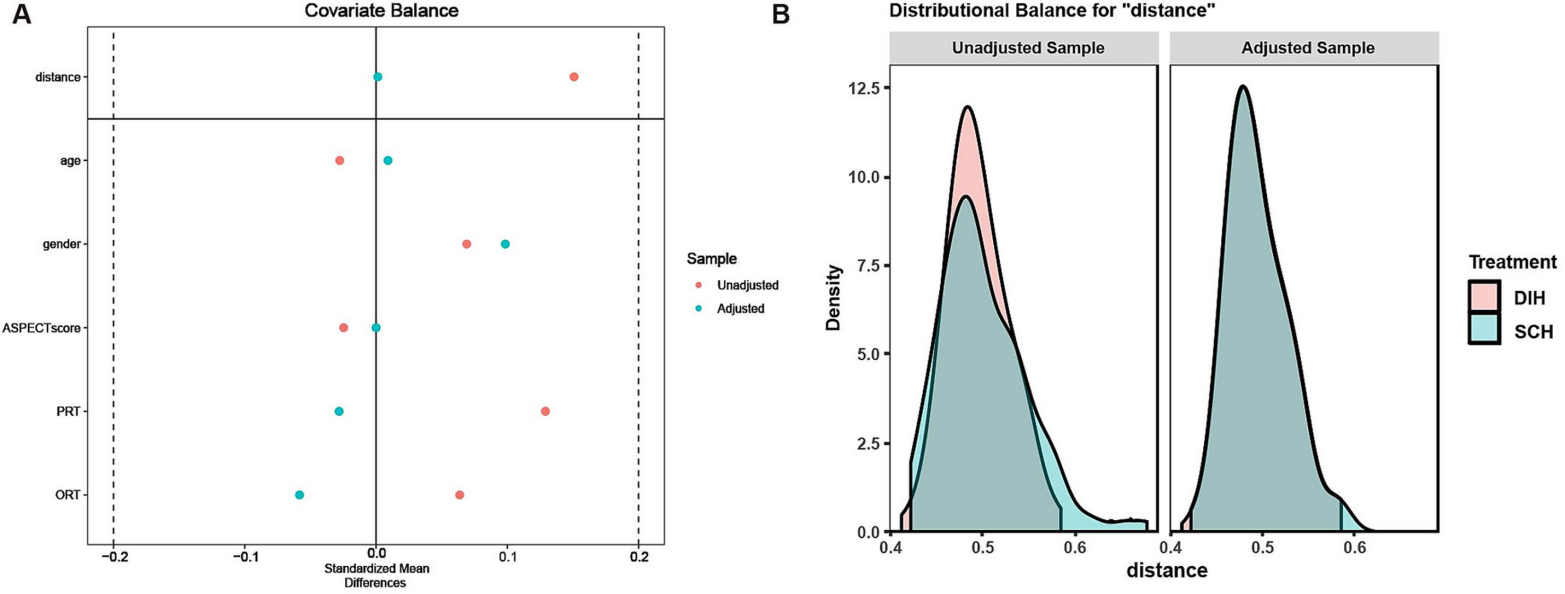
Balanced measures between the two groups after PSM. **(A)** Standardized mean differences for covariates included in propensity score matching in prematching (unadjusted) and postmatching (adjusted) samples. A standardized mean difference of <0.2 indicates well-matched samples. **(B)** Unadjusted (unmatched) and adjusted (matched) group comparison for DIH and SCH. Density overlay for “distance” shows good comparability after propensity score matching. DIH, deep intraparenchymal hyperdensities; SCH, subarachnoid/cortical hyperdensities. ASPECT, Alberta Stroke Program Early Computed Tomography Score; PRT, puncture to reperfusion time; ORT, onset to reperfusion time.

To balance confounding factors between the two groups, we matched the three variables above that were significantly different between the two groups, as well as demographics (age and gender). Finally, 116 patients in the DIH group were matched with 116 patients in the SCH group at a 1:1 ratio, with 52 unmatched patients in the DIH group and 22 in the SCH group. Post-PSM balance measures indicated a good match between the two groups (Figure 4). Comparison of the DIH group and the SCH group after PSM showed equal distribution of all variables (Table 1).

**Figure 5:**
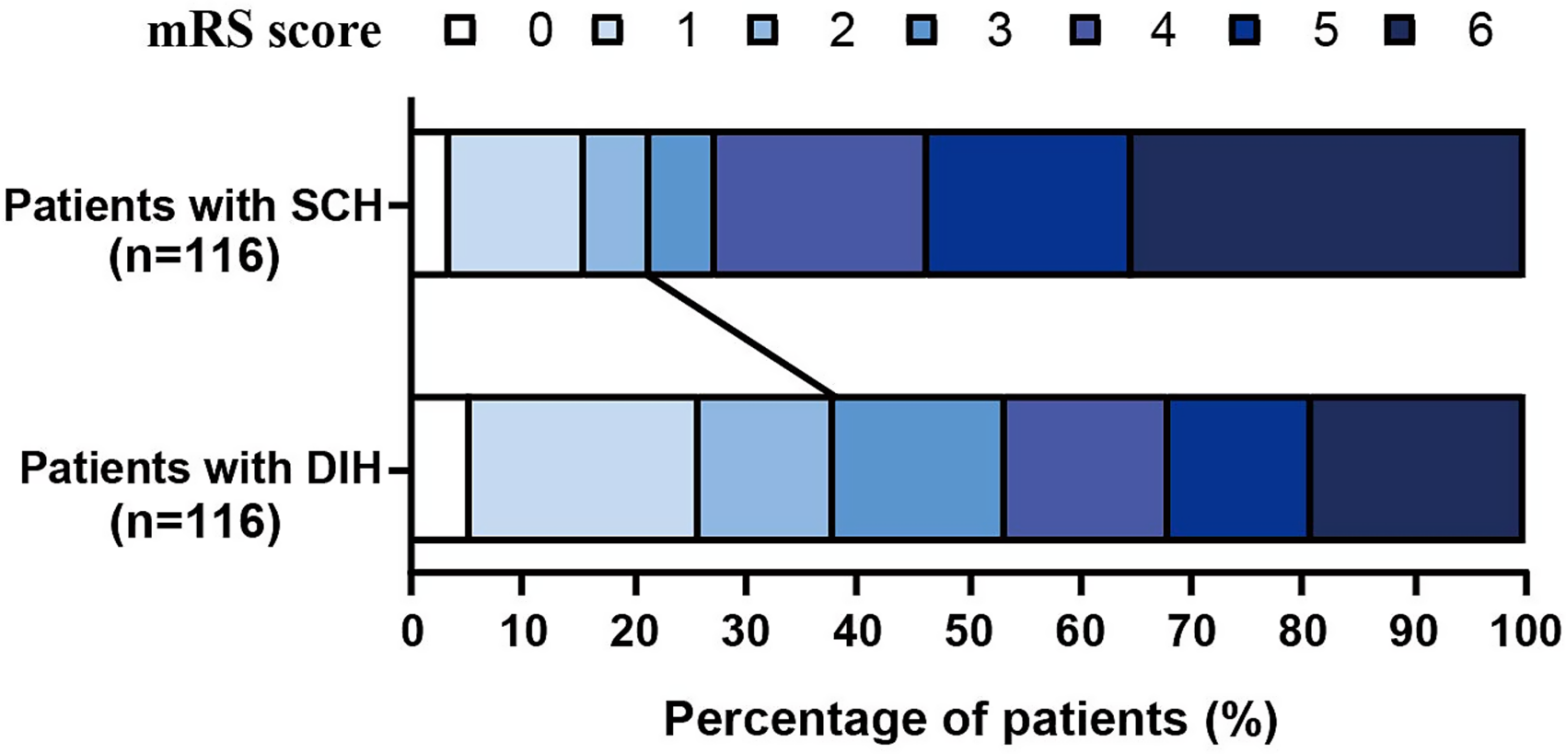
Distribution of the 3-month mRS scores for patients in the PSM cohort.

The distribution of the 3-month mRS scores for patients in the PSM cohort is shown in Figure 5. After PSM, 91 patients (78.5%) in the SCH group and 72 patients (62.1%) in the DIH group suffered FR (*p* = 0.006). After PSM, we performed univariate and multivariate analyses between the futile reperfusion and good reperfusion groups (Table 2). Eight variables were significantly different between the two groups, which were further analyzed using a univariate analysis. Finally, the multivariate logistic regression analysis derived a model, including NIHSS score, ASPECT score, ORT, and HDA types, to predict FR in patients with HDAs. The adjusted model revealed that, compared with the DIH group, the SCH group had a significantly elevated risk of FR (OR: 3.103, 95%CI: 1.425–6.759, *p* = 0.004), indicating that patients with SCH have a 3.103 times higher risk of FR than patients with DIH. To assess the robustness of the results, we conducted sensitivity analyses using IPTW to adjust for potential residual confounding. The IPTW-adjusted analysis yielded consistent findings: compared with the DIH group, the SCH group exhibited a significantly increased risk of FR (OR: 2.23, 95%CI: 1.35–3.69, *p* = 0.002, Table 3).

**Table 2:** Comparison between the good reperfusion and futile reperfusion groups in the PSM cohort.

Variables	Good reperfusion (*n* = 69)	Futile reperfusion (*n* = 163)	Univariate analysis		Multivariate analysis	
			OR (95%CI)	*P*-value	OR (95%CI)	*P*-value
Demographic data						
Age (years)	66.99 ± 12.39	70.61 ± 10.59*	1.029 (1.003–1.055)	0.026		
Male, *n* (%)	38 (55.1)	80 (49.1)				
Vascular risk factors, *n* (%)						
Diabetes mellitus	14 (20.2)	45 (27.6)				
Hypertension	41 (59.4)	103 (63.2)				
Hyperlipidemia	9 (13)	17 (10.4)				
Atrial fibrillation	43 (62.3)	98 (60.1)				
Coronary heart disease	6 (8.7)	16 (9.8)				
Previous stroke	9 (13)	10 (6.1)				
Smoking	15 (21.7)	37 (22.7)				
Alcohol consumption	18 (26.1)	37 (22.7)				
Stroke etiology, *n* (%)						
LAA	22 (31.9)	63 (38.7)				
CE	44 (63.8)	99 (60.7)				
Others	3 (4.3)	1 (0.6)				
Occlusive site, *n* (%)						
ICA	28 (40.6)	78 (47.8)				
MCA	41 (59.4)	85 (52.1)				
Admission baseline data						
SBP (mmHg)	146.99 ± 25.03	150.14 ± 24.30				
DBP (mmHg)	85.28 ± 16.04	84.37 ± 14.47				
NIHSS score	18 (14.5–20)	25 (22–28)*	1.273 (1.187–1.366)	<0.001	1.236 (1.142–1.338)	<0.001
ASPECT score	9 (8.5–10)	8 (6–8)*	0.425 (0.319–0.566)	<0.001	0.499 (0.362–0.688)	<0.001
MT treatment procedure						
Number of thrombectomy passes	2 (1–3)	2 (1–3)				
OPT (min)	264.36 ± 91.40	296.82 ± 105.71*	1.003 (1–1.006)	0.029		
PRT (min)	61 (42–87)	81 (57–118)*	1.014 (1.005–1.022)	0.001		
ORT (min)	338.2 ± 82.07	399.5 ± 97.95*	1.008 (1.004–1.012)	<0.001	1.007 (1.002–1.011)	0.008
Thrombolysis, *n* (%)	12 (17.4)	39 (23.9)				
mTICI score	5 (5–5)	5 (4–5)*	0.454 (0.269–0.766)	0.003		
HDA types, *n* (%)						
DIH	44 (37.9)	72 (62.1)*	Reference		Reference	
SCH	25 (21.6)	91 (78.4)*	2.224 (1.245–3.973)	0.007	3.103 (1.425–6.759)	0.004

Values are listed as frequency (percentage), mean ± SD, or median (interquartile range). The “*” indicates “*P* < 0.05” compared with the good reperfusion group. HDAs, hyperdense areas; DIH, deep intraparenchymal hyperdensities; SCH, subarachnoid/cortical hyperdensities; PSM, propensity score matching; SBP, systolic blood pressure; DBP, diastolic blood pressure; NIHSS, National Institute of Health Stroke Scale; ASPECT, Alberta Stroke Program Early Computed Tomography Score; LAA, large artery atherosclerosis; CE, cardioembolism; ICA, internal carotid artery; MCA, middle cerebral artery; OPT, onset to puncture time; PRT, puncture to reperfusion time; ORT, onset to reperfusion time; mTICI; modified thrombolysis in cerebral infarction. For statistical analysis, the mTICI score was numerically encoded as follows: 0 = 1, 1 = 2, 2a = 3, 2b = 4, and 3 = 5.

**Table 3:** Comparative analysis of FR risk prediction by SCH versus DIH across different statistical methods.

Model	Regression coefficient	OR	95%CI	*P*-value
Unmatched model	1.135	3.112	1.532–6.322	0.002
PSM-matched model	1.132	3.103	1.425–6.759	0.004
IPTW-adjusted model	0.894	2.231	1.353–3.692	0.002

PSM, propensity score matching; IPTW, inverse probability of treatment weighting.

## 4 Discussion

Currently, FR is becoming a major challenge in the endovascular treatment of patients with AIS and an emerging concern for neurointerventionalists (15). However, the pathophysiology of FR remains unclear. The underlying mechanisms of FR include the “no-reflow” phenomenon, initial tissue damage, reperfusion injury, cerebral edema, poor collateral flow, and inflammation (15, 16). Notably, reperfusion injury is an important mechanism of FR, and many studies have identified it as an independent risk factor for poor prognosis 3 months after MT (8, 17).

Previous studies have found a strong association between HDAs and reperfusion injury (8, 9), which may explain the higher rate of FR in patients with HDAs. Reported incidence rates of HDAs range from 31% to 84% in the literature (11, 18–20), with our study observing a rate of 66.4%. Consistent with existing evidence (10–12, 21), our findings confirm an increased risk of poor outcomes in patients with HDAs. In our study, FR occurred in 38.8% of patients without HDAs, with a significantly higher incidence (67.6%) observed among those with HDAs.

Post-thrombectomy HDAs in distinct intracranial compartments may represent different underlying pathophysiological mechanisms, which consequently demonstrate significant prognostic heterogeneity (22). Our study categorizes post-thrombectomy HDAs into two types (DIH and SCH) based on anatomical location. DIH is predominantly caused by microvascular compromise, manifesting as blood–brain barrier dysfunction and impaired microvascular integrity (13). These pathological changes can induce regional cerebral hypoperfusion, ultimately contributing to unfavorable neurological outcomes (23, 24). In contrast, SCH reveals a strong association with extensive reperfusion phenomena (14). In cases of severe vascular injury, excessive reperfusion may induce mechanical damage to the vascular wall, further compromising the blood–brain barrier and allowing macromolecules to extravasate into the brain tissue, exacerbating injury (25, 26). Additionally, this process may involve dual mechanisms of vascular and parenchymal damage (16), where inflammatory responses and vasospasm worsen cerebral ischemia, while elevated intracranial pressure reduces perfusion (27). Furthermore, SCH has been strongly associated with malignant cerebral edema, which may contribute to the poor prognosis observed in some patients (28, 29). Therefore, patients with SCH exhibit poorer clinical outcomes than those with DIH. Consistent with these findings, our results revealed that patients with SCH had a significantly higher risk of FR than those with DIH after PSM (OR: 3.103, 95%CI: 1.425–6.759, *p* = 0.004), indicating that patients with SCH had a 3.103-fold higher FR risk than those with DIH. This elevated risk may be mediated by more extensive inflammatory responses, vasospasm, and intracranial hypertension in patients with SCH.

There are several limitations in the present study. First, it was a single-center retrospective study, which may have some selection bias, and further multicenter prospective studies are needed to reduce bias. Second, functional outcomes at 3 months post-MT were associated with rehabilitation compliance and financial factors, which we were unable to examine due to the study limitations. Third, the relatively small sample size may limit the generalizability and statistical power of the findings. Future studies with larger samples are needed to validate these results.

## 5 Conclusion

This study elucidates the heterogeneous nature of post-thrombectomy HDAs through radiologic subtype classification. Our findings indicate that SCH patients have a significantly higher FR risk than DIH patients. We demonstrate a direct correlation between imaging subtypes and clinical outcomes, providing an objective basis for early risk stratification. These results offer critical guidance for optimizing perioperative management in neurointerventional therapy. Future multicenter prospective studies are warranted to validate these observations and explore subtype-specific therapeutic interventions.

## Data Availability

The raw data supporting the conclusions of this article will be made available by the authors, without undue reservation.
